# The Impact of Socio Economic Factors on the Adherence of Patients with Gestational Diabetes Mellitus to Medical Recommendations

**Published:** 2019-09

**Authors:** Ali Akbar HAGHDOOST, Mohammad Reza BANESHI, Alireza RAZZAGHI, Adel NOORI

**Affiliations:** 1. Social Determinants of Health Research Center, Institute for Futures Studies in Health, Kerman University of Medical Sciences, Kerman, Iran; 2. Modeling in Health Research Center, Institute for Futures Studies in Health, Kerman University of Medical Sciences, Kerman, Iran; 3. Safety Promotion and Injury Prevention Research Center, Shahid Beheshti University of Medical Sciences, Tehran, Iran

**Keywords:** Gestational diabetes mellitus, Socio-economic status, Adherence, Medical orders, Factor analysis

## Abstract

**Background:**

Adherence to medical recommendations is very important to control gestational diabetes mellitus (GDM), as one of the most important disorders during pregnancy. In this study, we explored the impact of socio-economic status (SES) on the adherence of a cohort of GDM in Iran.

**Methods:**

In this prospective study, 230 pregnant women with confirmed GDM were followed from Feb to Jun 2013 in a referral diabetic care center in Iran. The SES of subjects were quantified using a combined score generated by principal component analysis (PSA). Medical adherence score of subjects was measured in three follow up visits in a range of 0 to 10 and were linked to SES using linear regression model.

**Results:**

The adherence scores women in the first, second, and third follow up visits were 5.06±2.12, 5.46±2.06, and 5.08±1, respectively. Women fourth quartile of SES (the highest level of SES) has a least compliance to medical orders in comparison to first quartile of SES (the lowest level of SES) with the OR −2.75 (95% Cl: −3.17, −2.23).

**Conclusion:**

The medical adherence of pregnant women with GDM is significantly poorer in high SES groups. Therefore, as an important determinant, we may target high SES pregnant women to control the adverse effects of the disorder more efficiently.

## Introduction

The gestational diabetes mellitus (GDM) is a disorder related to tolerating the carbohydrates diagnosed in pregnancy for the first time and is one of the most rampant complications of pregnancy (1, 2). The GDM can affect proportion of women during the pregnancy period. The prevalence of GDM ranges from 3% to 5%, and it is a known risk factor for type 2 diabetes after the pregnancy (3, 4). The prevalence of GDM has increased universally over the recent decade, which can relevant on lifestyle and non-awareness about the disease. Therefore, there is need to pay more attention to management the disease and reduce the consequences of that(5).

To management the diseases and reducing the risks of health complications, there are some treatments for women who suffer GDM in pregnancy period. One of the main issues in treatment is adherence to it. Nonadherence to treatment has a social and economic affect. Therefore, it has a considerable economic burden due to preventable complications and poor disease outcomes (6, 7). Women with GDM are at risk of type 2 diabetes mellitus and more likelihood of recurrence of the GDM in next pregnancies. Moreover, babies of GDM women are at risk of stillbirth and some complications such as; macrosomia, prematurity, hypoglycemia, and respiratory distress syndrome (8, 9).

There are some factors, like SES, can have a considerable effect on adherence to chronic drug regimens (10, 11). Compatible with the comprehensive definition of WHO, health is influenced by the socio-economic factors in addition to genetic and biologic factors (12). Social factors play an important role in the adherence of patients to medical recommendations (12). Moreover, factors such as the levels of education, occupation, income, housing, nutrition, environment, workplace, poverty, water, unemployment, stress, culture, etc. have significant direct and indirect roles on the health status of individuals and communities (13).

There are some evidence about the influencing the SES on adherence to treatment among diabetic patients. For example, low level of SES can affect the outcomes of diabetic patients, which consequently, has relationship with mortality, and complications of disease (14–16). The importance of GDM is far greater than another type of diabetes because of the health status of mother and child. Women with GDM are at high risk of some complications during and after pregnancy. The babies of GDM women are threatened with various defects and complications too (8,9), which some of these outcomes can relate to SES. However, there is a lack of sufficient information about the effects of socio-economic factors on the level of gestational diabetic patients’ adherence to medical treatment. Identifying the SES role in affecting GDM management can help medical care team to providing better medical care, which can be beneficial for the health of both mother and child.

We aimed to explore the relationship between SES and adherence of patients with GDM to medical orders.

## Methods

This prospective study was carried out from Feb to Jun 2013. The population of the study included women with GDM who were in the sixth month of their pregnancy or after that and had referred to diabetes medicinal center located in Bahonar Hospital of Kerman, Iran for pregnancy control during the study period.

Overall, 230 individuals referred to the only referral center for pregnancy care, participated in the study. The eligibility criteria included mothers who were Iranian, mothers without any problems affecting their pregnancy and mothers who, based on their own words, had no physical or mental illnesses, or addiction to cigarette, alcohol, or any other drugs.

Data were collected through the medical profiles of participants and administration of questionnaire.

The questionnaire consisted of our main parts, first demographic questions (age, height, weight, pregnancy age, pregnancy rank); second questions about illnesses and case history (hypertension, high edema of hands and feet, consciousness disorder, severe passion, urinary infection, diabetes, diabetes type 2 in relatives, pregnancy diabetes). The third part includes questions regarding socio-economic factors (education, occupation, income, the number of employed people in the family, the number of family members, the possession of different properties), and the last part of the questions was about adherence of patients to medical instructions (eating snacks during the day, regular monitoring of blood sugar, the number of insulin injection, eating vegetables and fruits, and doing exercise during the week).

The questions regarding the adherence of the patients to medical orders were answered at least once and at most three times every two weeks and finally; a total score was obtained for each person by adding all scores considered as their adherence score. In general, the range of scores was 0 to 10. In order to obtain a general variable and a total index for SES variable, all individuals were asked questions about their SES. These questions were about the education level of pregnant women and their husbands, her job and her husband’s, residential area, number of family members, number of the employed individuals in their family, income, number of the rooms, number of trips out of the city, their properties (the type of possession, personal car, motorcycle, bicycle, LCD TV, computer, laptop).

Content validity was used in order to get the scientific validity of the collection tools. The questionnaire was submitted to some members of faculty of the Kerman University of Medical Science authorized in diabetes and socio-economic issues. Having applied the recommended modifications, content of the questionnaire was evaluated and confirmed. In order to study the reliability of the questionnaire, the Cronboch’s Alpha method was used and alpha coefficient was calculated to be 0.79%.

Using Principal Component Analysis (PCA), 9 variables related to the measurement of the SES including income, education, occupation, and possession of different properties were selected for further analysis. The Vairmax technique was used in order to make a variable called socio-economic level, and ultimately the final variable of SES was made. Therefore, a factor score was given to each patient using regression method indicating their rank of socio-economic level so that patients were ultimately divided into 4 groups according to their score which was a quantitative variable by using cut points and were belonged to one of these quartiles as a qualitative variable. First quartile shows the initial 25% of individuals with lower SES status and fourth quartile represents the last 25% of people with higher SES status (17).

The linear regression test was used as mono-variable and multivariable in order to determine the effect of socio-economic index on patients’ adherence to medical orders. All statistical analyses were done using SPSS 18 (Chicago, IL, USA), Stata 12, and the level of *P*<0.05 was considered for statistical significance.

### Ethical approval

The Ethical Committee of Kerman University of Medical Sciences approved the present study and its protocol.

## Results

84.8% of the patients (N=195) were under 35 yr old. In considering the SES, 19.1% (N=44) of women and 27% (N=62) of their husbands had education lower than high school. About 80% (N=184) of women and 16.5% (N=38) of their husbands were in professional group including, worker, farmer, housewife, and university student. In terms of residential area, 40.9% (N=94) of patients were living in areas with low levels of economy. In terms of income, 56.1% (N=129) of patients had low income, 35.2% (N=81) were in middle class, and 8.7% (N=20) had high levels of income. Table 1 shows the demographic data and clinical features of 230 with GDM. Regarding the birth order, 42.2% (N=97) of pregnant women were ranked first, 35.2% (N=81) were ranked second, and 22.6% (N=52) were ranked third or higher.

**Table 1:** Descriptive and demographic data of patients studied

***Variable***	***Frequency***	***Percentage***	***Variable***	***Frequency***	***Percentage***
Age	Urinary infection				
<35 yr	195	84.8	No	167	72.6
<35 yr	35	15.2	Yes	63	27.4
Birth Order	Type 2 diabetes				
1	97	42.2	No	194	84.3
2	81	35.2	Yes	36	15.7
3 and above	52	22.6			
Months of pregnancy	History of GDM*				
6	43	18.7	Yes		
7	59	25.6	No	22	9.6
8	86	37.4		208	90.4
9	42	18.3			
Blood pressure	Control type 2 diabetes before pregnancy				
No	197	85.7	Modification of life style Oral drugs	25	10.9
Yes	33	14.3		8	3.5
			Injecting drugs	3	1.3
High edema hand and feet			History of type 2 diabetes in relatives		
No	172	74.8	Yes	116	50.4
Yes	58	25.2	No	114	49.6
Disorder of consciousness			The blood sugar before pregnancy		
			Under control		
No	224	97.4	Controlled	13	5.7
Yes	6	2.6	I do not know	19	8.3
				4	1.7

* -Gestational Diabetes Mellitus

In the (PCA), two factors were discovered: 1) which predicted 43% of total variance and was formed by seven variables such as laptop, LCD TV, income, education of pregnant women’s husbands, education of pregnant women, residential area, trips outside of the city, and the occupation of pregnant women’s husband; 2) In second identified factor there were two variables: number of employed individuals and the occupation of pregnant women. In other words, individuals who were in the lower quartile had better adherence to medical orders than those who were in upper quartiles (*P*-value<0.001).

The socioeconomic variable justifies 43% of the changes in adherence variable (R^2^: 0.43). The mean score of adherence for individuals with lower education is better than those with higher education (Table 2).

**Table 2:** The effect of socioeconomic and clinical factors on adherence to prescription using the linear regression model

***Variable***	***Univariate***			***Multivariate***		
	***β (95% CI)***	***P-value***	***R^2^***	***β (95% CI)***	***P-value***	***R^2^***
Education of women						0.52
<High school(Ref)	-	-	0.23	------	------	
High school	−0.53 (−1.04, −0.02)	0.004		−0.02 (−0.48, 0.44)	0.922	
>High school	−1.92 (−2.43, −1.41)	<0.001		−0.71 (−1.23, −0.19)	0.007	
Education of men						
<High school(Ref)	-		0.15	------	------	
High school	−1.10(−1.59, −0.62)	<0.001		−0.23 (−0.64,0.17)	0.267	
>High school	−1.61(−2.11, −1.11)	<0.001		0.09 (−0.40, 0.58)	0.719	
Occupation position (women)						
First (Professional,ref)	---	---	0.20	------	------	
2th and 3th (Skilled)	−1.82(−2.29, −1.35)	<0.001		−1.16 (−1.53, −0.79)	<0.001	
Occupation position (men)						
First (Professional, ref)	---	---	0.15	------	------	
2th (Skilled workers Semi-skilled/unskilled)	−.045(−0.60, 0.51)	0.870		0.27 (−0.13,0.68)	0.187	
3th (Semi-skilled/unskilled)	−1.34(−1.91, −0.77)	<0.001		−0.43 (−0.91, 0.03)	0.067	
Residential Area						
Low (Ref)	---	---	0.24	------	------	
Medium	−0.97(−1.37, −0.56)	<0.001		−0.64 (−1.08, −0.19)	0.005	
High	−2.2(−2.73, −1.70)	<0.001		−1.04 (−1.66, −0.41)	0.001	
Income level						
Low (Ref)	---	---	0.21	------	------	
Medium	−0.52(−1.22, 0.18)	0.140		−0.20 (−0.81,0.40)	0.505	
High	−1.91(−2.59, −1.23)	<0.001		−0.23 (−0.97, 0.49)	0.524	
SES variable*						
1st quartile (the lowest, ref)	---	---	0.43	---	---	
2nd quartile	−0.80(−1.25, −0.35)	<0.001		−0.55 (−0.94, −0.15)	0.006	
3rd quartile	−1.51(−1.96, −1.06)	<0.001		−1.2 (−1.65, −0.85)	<0.001	
4th quartile (the highest)	−2.87(−3.32, −2.42)	<0.001		−2.75 (−3.17, −2.33)	<0.001	
Birth Order	---	---	---			
1 (Ref)	---	---	---	------	------	
2	---	---	---	1.27 (0.96, 1.59)	<0.001	
3 and above	---	---	---	−0.05 (−0.42, 0.31)	0.763	
High edema hand and feet	---	---	---			
No (Ref)	---	---	---	------	------	
Yes	---	---	---	−0.15 (−0.51, 0.20)	0.401	
Type 2 diabetes	---	---	---			
No (Ref)	---	---	---	------	------	
Yes	---	---	---	−0.02 (−0.50, 0.45)	0.913	

* SES= Socio-economic Status

## Discussion

The aim of this study was studying the effects of SES on the adherence of patients suffering from pregnancy diabetes to medical orders. The most important findings of this study are as follows: the total mean score of adherence was 4.89 (maximum score was 10). According to the results of multivariable analysis, and after checking the influence of another variable (by removing the influence of possible defacing variables), the SES was identified as an effective factor.

The relationship between SES and adherence to medical orders can be considered from different aspects. The role of economic problems in providing the medication and treatment requirements recommended by the doctor on the one hand and the influence of cultural factors mentioned in Stasenko’s study, on the other hand (18).

In the case of education, the most important factors that can be the justifiers of its relationship with the adherence to medical orders would be important role of individual’s lack of knowledge in following the instructions given by the people with higher knowledge. These individuals refer more regularly to the health care centers, which lead them to correct way of treatment and help them follow medical orders. On the other hand, individuals with higher education do not accept any kind of treatment and sometimes they need to be informed about their treatment.

In our study, women in areas with low socioeconomic status have a higher level of compliance in comparison to another residential area. One explanation for that is occupational position of women. Therefore, women in low level of socioeconomic status are generally housewives or have a job that does not take much of their lives. Being employed and busy were the main factors in non-compliance of diabetes patients with treatment (14).

Generally, the main finding of our study is that women with low level of SES have better compliance with medical orders in comparison to other women. This finding is unexpected and suggested that pregnant women with high level of socioeconomic status have lower compliance to medical orders. Possible explanation for this is that pregnant women with low level of education have high risk of perception (14). Therefore, they try to adhere to medical treatment in order to avoid the unfavorable physical and economic consequence. However, further research is needed to investigate this issue.

Those who have high education are usually employed and this can lead to lack of attention to their health status. Moreover, in some patients, fear of insulin treatment was one of the issues, which may contribute to non-adherence of medical treatment (19). Non-adherence to medical treatment is acceptable behavior in social and cultural groups. In this situation, adherence to medical treatment may lead to placing oneself outside of the group (20).

## Conclusion

The adherence of patients with GDM to medical orders is a multifactorial phenomenon. Multilateral programming is needed in order to increase the amount of this adherence and general management programs should be applied without removing any possible factors in an attempt to study the obtained results about the inferences of augmenter of adherence for considering all effective factors and their effects. Adherence to the medical orders in patients with GDM has a significant relationship with socio-economic factors. Therefore, we can try to increase the adherence of patients with GDM by educating target groups and doing social interventions. While some people, especially those who are in high level of SES may receive the essential pregnant period cares from private sector rather than diabetes medicinal center, there may be slight selection bias.

## Ethical considerations

Ethical issues (Including plagiarism, informed consent, misconduct, data fabrication and/or falsification, double publication and/or submission, redundancy, etc.) have been completely observed by the authors.
